# METTL3 stabilizes HDAC5 mRNA in an m^6^A-dependent manner to facilitate malignant proliferation of osteosarcoma cells

**DOI:** 10.1038/s41420-022-00926-5

**Published:** 2022-04-08

**Authors:** Renbing Jiang, Zhibing Dai, Junshen Wu, Suzhi Ji, Yachao Sun, Wenpeng Yang

**Affiliations:** Department of Bone and Soft Tissue, Affiliated Tumor Hospital of Xinjiang Medical University, 830011 Urumqi, Xinjiang China

**Keywords:** Mechanisms of disease, Cancer therapy

## Abstract

Osteosarcoma (OS) is a prevalent primary bone sarcoma. Methyltransferase-like 3 (METTL3) is dysregulated in human malignancies. This study explored the mechanism of METTL3 in OS cell proliferation. Our results demonstrated that METTL3 was highly expressed in OS, and correlated with the tumor size, clinical stage, and distant metastasis of OS patients. Higher METTL3 expression indicated poorer prognosis. METTL3 silencing inhibited the malignant proliferation of OS cells, while METTL3 overexpression led to an opposite trend. METTL3 upregulated histone deacetylase 5 (HDAC5) expression in OS cells by increasing the m^6^A level. HDAC5 reduced the enrichment of H3K9/K14ac on miR-142 promoter, thus suppressing miR-142-5p expression and upregulating armadillo-repeat-containing 8 (ARMC8) level. HDAC5 overexpression or miR-142-5p silencing attenuated the inhibitory effect of METTL3 silencing on OS cell proliferation. Xenograft tumor experiment in nude mice confirmed that METTL3 silencing repressed OS cell proliferation in vivo via the HDAC5/miR-142-5p/ARMC8 axis. Collectively, METTL3-mediated m^6^A modification facilitated OS cell proliferation via the HDAC5/miR-142-5p/ARMC8 axis.

## Introduction

Osteosarcoma (OS), the most prevalent primary solid bone malignancy, is defined as the production of osteoid and/or immature bone by malignant mesenchymal cells [1]. The etiology of OS is still largely unknown, but OS tends to occur at the age of puberty growth spurt and the site of maximum growth, suggesting the association between OS and rapid bone proliferation [2]. The typical manifestations of OS include severe pain and swelling of the affected bones, and OS patients occasionally develop other symptoms related to pathological fractures [3]. Lung metastasis and subsequent relapse remain the prominent causes of clinical death in OS patients [4]. Therefore, it is imperative to explore potent targets to restrain OS cell malignant proliferation for the improvement of clinical outcomes.

N^6^-methyladenosine (m^6^A), a type of post-transcriptional modification of RNA, represents the most prevalent internal modification of mRNAs [5]. m6A modification is installed by m^6^A methyltransferases (termed as “writers”), reverted by demethylases (termed as “erasers”), and recognized by m^6^A binding proteins (termed as “readers”) [6]. m^6^A modification is implicated in diverse physiology and pathology by modulating RNA stability, mRNA translation, and microRNA (miRNA) processing [7]. Compelling evidence has unveiled that altered m^6^A level contributes to tumor initiation and progression by controlling tumor-related genes [8]. m^6^A-related regulators have been documented to be dysregulated in OS patients, which are accepted as promising therapeutic targets and prognostic indicators of OS [9]. Methyltransferase-like 3 (METTL3) is identified as a pivotal methyltransferase responsible for catalyzing m^6^A formation [10]. A recent study has demonstrated the upregulated METTL3 expression and m^6^A level in OS tissues and cells [11]. METTL3 exerts a promoting effect on OS progression, and METTL3 deficiency retards the proliferative capacity of OS cells [12]. However, the exact mechanism of METTL3 in the malignant proliferation of OS cells has not been thoroughly clarified.

Histone deacetylases (HDACs), a family of enzymes, catalyze the removal of the acetyl group from the lysine residues of histones, and overexpression of HDACs serves as a notable indicator of dismal outcomes in OS [13]. METTL3 is reported to induce the osteogenic differentiation of bone marrow mesenchymal stem cells by modulating its target HDAC5 through m^6^A modification [14]. HDAC5 belongs to the class II HDAC subfamily and regulates gene transcription through histone acetylation and chromatin remodeling [15]. HDAC5 expression is dramatically elevated in OS cells, and HDAC5 overexpression expedites OS cell proliferation [16]. Accordingly, we infer whether METTL3 can mediate HDAC5 expression through m^6^A modification to participate in OS cell proliferation. This study set out to investigate the function of METTL3-mediated m^6^A modification in OS cell proliferation and determine the potential downstream mechanism of METTL3/HDAC5 in OS cells, hoping to uncover a novel target for OS treatment.

## Results

### METTL3 expression was elevated in OS and correlated with prognosis and clinicopathological features of OS patients

**Fig. 1:**
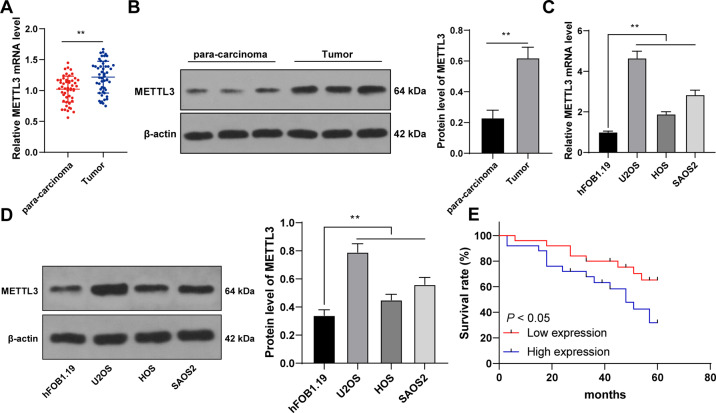
METTL3 was highly expressed in OS tissues and cells and correlated with the prognosis and clinicopathological features of OS patients. **A**, **B** METTL3 expression in 50 pairs of OS tissues and adjacent tissues was detected using RT-qPCR and western blot. **C**, **D** METTL3 expression in human OS cell lines (U2OS, HOS, SAOS2) and normal osteoblasts (hFOB1.1) was detected using RT-qPCR and western blot. **E** With the median mRNA level of METTL3 in OS tissues as the critical value, the correlation between METTL3 mRNA level and prognosis of 50 OS patients was analyzed using Kaplan–Meier survival curve. *N* = 50. The cell experiment was repeated three times independently. Data in panels **B**–**D** are presented as mean ± standard deviation. Data in panel **A** were analyzed using paired *t*-test and data in panel **B** were analyzed using *t*-test. Data comparisons between two groups in panel **E** were analyzed using Log-Rank test and data comparisons among multiple groups in panels **C**, **D** were analyzed using one-way ANOVA, followed by Tukey’s multiple comparisons test, **p < 0.01.

METTL3-mediated m^6^A modification participates in the initiation and progression of multiple tumors [6, 17, 18]. OS patients present elevated METTL3 expression [11, 12, 19]. We aimed to investigate the specific effect of METTL3 on OS cell proliferation. METTL3 expression in 50 pairs of OS tissues and adjacent tissues was detected, and it was observed that METTL3 expression in OS tissues was much higher than that in adjacent tissues (*p* < 0.01, Fig. 1A, B). Similarly, METTL3 expression in OS cells was also higher than that in normal osteoblasts (*p* < 0.01, Fig. 1C, D). To explore the clinical significance of METTL3 in OS, we assigned the 50 OS patients into METTL3 high expression group and low expression group with the median METTL3 mRNA expression in OS tissues as the critical value [20]. Our results exhibited that METTL3 expression was correlated with tumor size, clinical stage, and distance metastasis (*p* < 0.05, Supplementary Table 2), and higher METTL3 expression indicated worse overall survival rate (*p* < 0.05, Fig. 1E). Briefly, METTL3 expression was enhanced in OS and correlated with the prognosis and clinicopathological features of OS patients.

### METTL3 silencing suppressed OS cell malignant proliferation

**Fig. 2:**
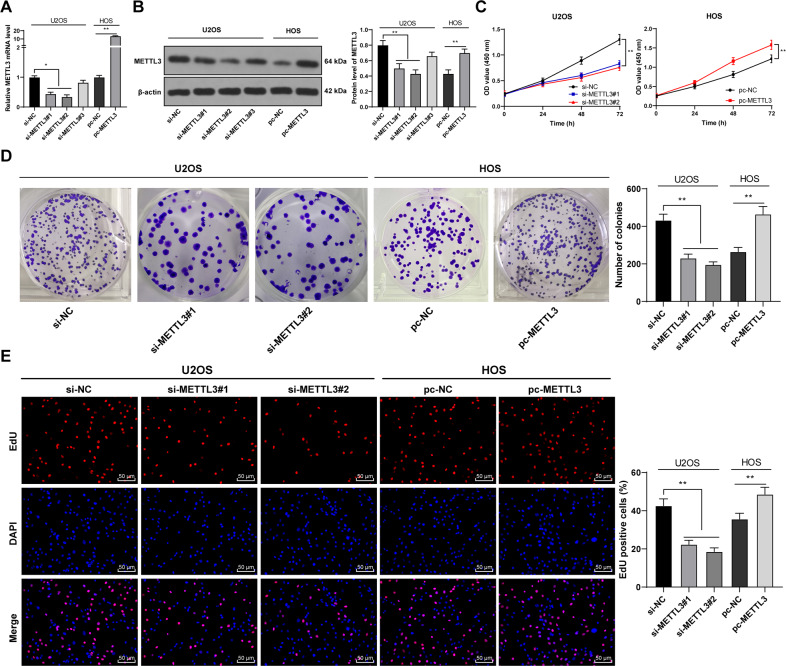
METTL3 silencing suppressed malignant proliferation of OS cells. Three Three METTL3 siRNAs (si-METTL3#1, si-METTL3#2, and si-METTL3#3) were transfected into U2OS cells with relatively high METTL3 expression, with si-NC as negative control. pcDNA 3.1 METTL3 (pc-METTL3) was transfected into HOS cells with relatively low METTL3 expression, with pc-NC as negative control. After 48 h, **A**, **B** METTL3 expression in OS cells was detected using RT-qPCR and western blot. Two siRNAs with high transfection efficiency (si-METTL3#1 and si-METTL3#2) were selected for subsequent experimentation. **B**–**D** The proliferation of OS cells was measured using CCK-8 assay (**B**), colony formation assay (14 days) (**C**), and EdU staining (**D**). The cell experiment was repeated three times independently. Data are presented as mean ± standard deviation. Data in panels **A**, **B**/**D**–**E** were analyzed using one-way ANOVA, and data in panel **C** were analyzed using two-way ANOVA, followed by Tukey’s multiple comparisons test, *p < 0.05, **p < 0.01.

To determine the function of METTL3 in OS proliferation, we intervened METTL3 expression in OS cells. Three METTL3 siRNAs (si-METTL3#1, si-METTL3#2, and si-METTL3#3) were transfected into U2OS cells with relatively high METTL3 expression, which successfully downregulated METTL3 expression in U2OS cells. Two siRNAs with high transfection efficiency (si-METTL3#1 and si-METTL3#2) were selected for subsequent experimentation. Moreover, pcDNA 3.1 METTL3 (pc-METTL3) was transfected into HOS cells with relatively low METTL3 expression, which successfully upregulated METTL3 expression in HOS cells (*p* < 0.05, Fig. 2A, B). METTL3 silencing notably reduced OS cell proliferation, while METTL3 overexpression enhanced OS cell proliferation (*p* < 0.01, Fig. 2C, D). EdU staining further validated the trends above (*p* < 0.01, Fig. 2E). Briefly, METTL3 silencing suppressed malignant proliferation of OS cells.

### METTL3 silencing reduced m^6^A modification in OS cells and thereby suppressed HDAC5 expression

**Fig. 3:**
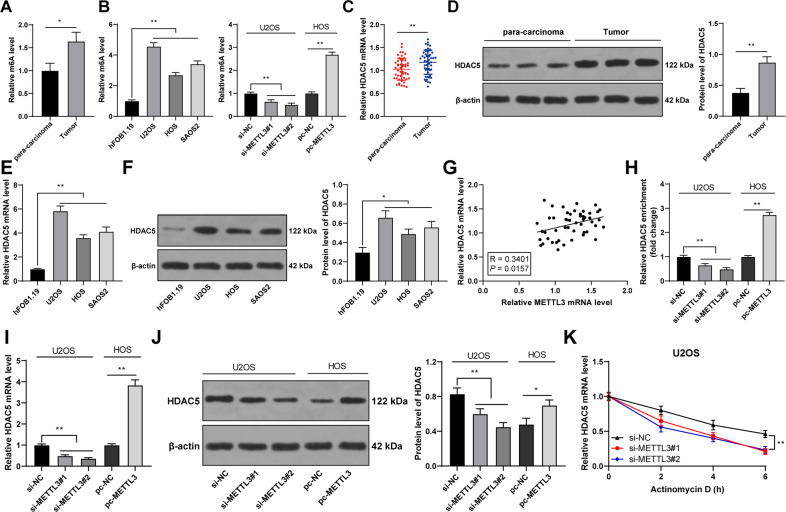
METTL3 silencing reduced m^6^A modification in OS cells and thereby suppressed HDAC5 expression. **A**, **B** m^6^A level in 50 pairs of OS tissues and adjacent tissues was detected by m^6^A quantitative analysis. **B** m^6^A level in human OS cell lines (U2OS, HOS, SAOS2) and normal osteoblasts (hFOB1.1) was detected by m^6^A quantitative analysis. **E**, **F** HDAC5 expression in human OS cell lines (U2OS, HOS, SAOS2) and normal osteoblasts (hFOB1.1) was determined using RT-qPCR and western blot. **G** The correlation between HDAC5 and METTL3 expression in OS tissues was analyzed using Pearson correlation analysis. **H** The m^6^A modification level of HDAC5 mRNA in OS cells after intervention with METTL3 expression was detected using MeRIP-qPCR. **I**–**K** HDAC5 expression and stability in OS cells after intervention with METTL3 expression was measured using RT-qPCR and western blot. *N* = 50. The cell experiment was repeated three times independently. Data in panels **A**, **B**/**D**–**F**/**H**–**K** are presented as mean ± standard deviation. Data in panel **C** were analyzed using paired *t*-test and data in panels **A**/**D** were analyzed using *t*-test. Data in panels **B**/**E**–**F**/**H**–**J** were analyzed using one-way ANOVA, and data in panel **K** were analyzed using two-way ANOVA, followed by Tukey’s multiple comparisons test, *p < 0.05, **p < 0.01.

Thereafter, we focused on the downstream mechanism of METTL3. METTL3 can affect HDAC5 expression through m^6^A modification [14], and HDAC5 expression is enhanced in OS [16]. Hence, we speculated that METTL3-mediated m^6^A modification regulated HDAC5 expression in OS. Our results exhibited that OS tissues and cells had elevated m^6^A level, while METTL3 silencing reduced m^6^A level and METTL3 overexpression led to an opposite trend (*p* < 0.05, Fig. 3A, B). HDAC5 expression was elevated in OS tissues and cells (*p* < 0.05, Fig. 3C–F), and METTL3 expression was positively correlated with HDAC5 expression in OS tissues (*p* < 0.01, Fig. 3G). METTL3 silencing notably reduced m^6^A modification level on HDAC5 mRNA and also decreased HDAC5 expression, while METTL3 overexpression had the opposite results (*p* < 0.01, Fig. 3H–J). Also, HDAC5 mRNA stability was attenuated after METTL3 silencing (*p* < 0.01, Fig. 3K). Briefly, METTL3 silencing reduced the m^6^A modification of HDAC5 in OS cells and suppressed HDAC5 mRNA stability, thereby decreasing HDAC5 expression.

### HDAC5 overexpression reversed the effect of METTL3 silencing on OS cell proliferation

**Fig. 4:**
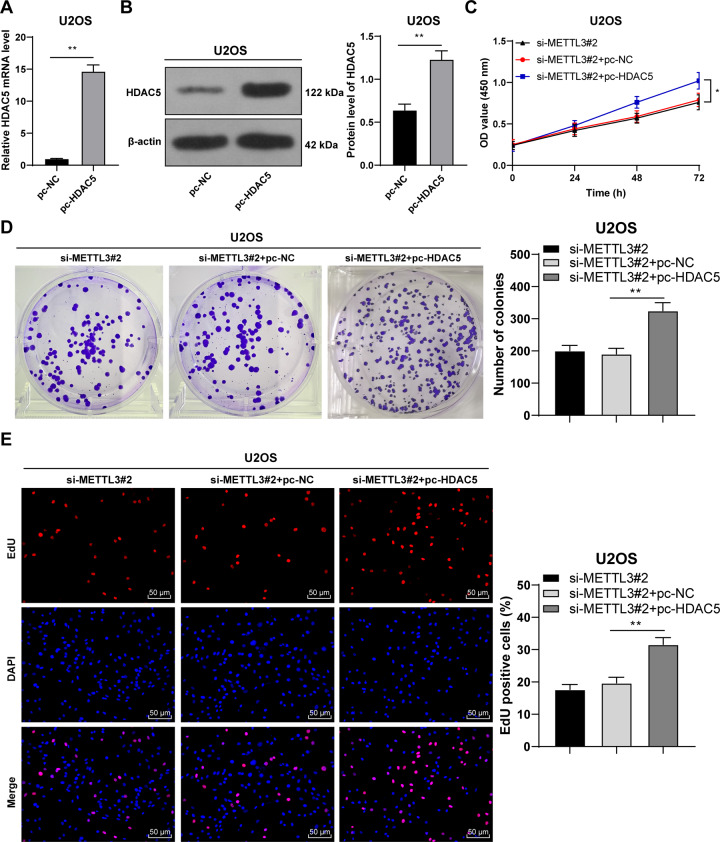
HDAC5 overexpression partially reversed the inhibitory effect of METTL3 silencing on OS cell proliferation. pcDNA 3.1 HDAC5 (pc-HDAC5) was transfected into U2OS cells, with pc-NC as negative control. After 48 h, **A**, **B** HDAC5 expression in U2OS cells was measured using RT-qPCR and western blot. Then, pc-HDAC5-transfected U2OS cells were treated with si-METTL3#2 for 48 h. **C**–**E** The proliferation of OS cells was measured using CCK-8 assay (**C**), colony formation assay (14 days) (**D**), and EdU staining (**E**). The cell experiment was repeated three times independently. Data are presented as mean ± standard deviation. Data in panels **A**, **B** were analyzed using *t*-test. Data in panels **D**, **E** were analyzed using one-way ANOVA, and data in panel **C** were analyzed using two-way ANOVA, followed by Tukey’s multiple comparisons test, *p < 0.05, **p < 0.01.

Next, we conducted a combined experiment to explore the role of HDAC5 in OS cell proliferation. Transfection of pcDNA 3.1 HDAC5 (pc-HDAC5) successfully upregulated HDAC5 expression in U2OS cells (*p* < 0.05, Fig. 4A, B). Then, the pc-HDAC5-treated U2OS cells were transfected with si-METTL3#2. Compared with METTL3 silencing alone, the combined treatment of HDAC5 overexpression + METTL3 silencing notably enhanced OS cell proliferation (*p* < 0.05, Fig. 4C, D). Briefly, HDAC5 overexpression partially attenuated the inhibition of METTL3 silencing on OS cell proliferation.

### HDAC5 reduced the acetylation of miR-142 promoter and thereby depressed miR-142-5p expression

**Fig. 5:**
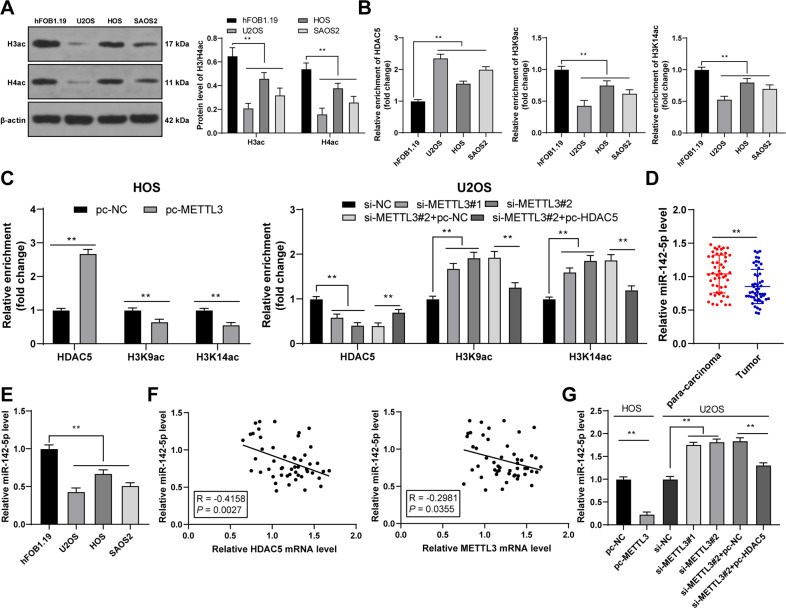
HDAC5 reduced the acetylation of miR-142 promoter and thereby inhibited miR-142-5p expression. **A** H3/H4ac level in human OS cell lines (U2OS, HOS, SAOS2) and normal osteoblasts (hFOB1.1) was detected using western blot. **B**, **C** The enrichment of HDAC5 and H3K9/K14ac on miR-142 promoter in human OS cell lines (U2OS, HOS, SAOS2) and normal osteoblasts (hFOB1.1) was analyzed using ChIP-qPCR. **D** miR-142-5p expression in 50 pairs of OS tissues and adjacent tissues was determined using RT-qPCR. **E** miR-142-5p expression in human OS cell lines (U2OS, HOS, SAOS2) and normal osteoblasts (hFOB1.1) was determined using RT-qPCR. **F** The correlation between miR-142-5p and HDAC5 and METTL3 in OS tissues was analyzed using Pearson correlation analysis. **G** miR-142-5p expression in OS cells after intervention with METTL3 and HDAC5 expression was measured using RT-qPCR. *N* = 50. The cell experiment was repeated three times independently. Data in panels **A**–**C**/**E**/**G** are presented as mean ± standard deviation. Data in panel **D** were analyzed using paired *t*-test. Data in panels **B**/**E**/**G** were analyzed using one-way ANOVA, and data in panels A/C were analyzed using two-way ANOVA, followed by Tukey’s multiple comparisons test, *p < 0.05, **p < 0.01.

The mechanism downstream of HDAC5 was explored. As a histone deacetylase, HDAC5 regulates the acetylation level of H3/H4 (H3/H4ac) [21]. Therefore, we detected the level of H3/H4ac in OS cells and found that the level of H3/H4ac was reduced in OS cells (*p* < 0.01, Fig. 5A). In addition, HDAC5 can act on miR-142 promoter and reduce the acetylation level of H3K9/K14, thereby reducing miR-142-5p expression [22]. miR-142-5p is poorly expressed in OS [23]. Therefore, we speculated that HDAC5 affected miR-142-5p expression by modulating the acetylation level of H3K9/K14 on miR-142 promoter. ChIP-qPCR results exhibited that HDAC5 and H3K9/K14ac were enriched on miR-142 promoter in OS cells (*p* < 0.01, Fig. 5B). Overexpression of METTL3 enhanced the enrichment of HDAC5 on miR-142 promoter and reduced the enrichment of H3K9/K14ac, while METTL3 silencing led to the opposite trends; after overexpression of HDAC5, the enrichment of HDAC5 on miR-142 promoter was enhanced and the enrichment of H3K9/K14ac was reduced (*p* < 0.01, Fig. 5C). In addition, miR-142-5p expression was declined in OS tissues and cells (*p* < 0.01, Fig. 5D, E), and there existed negative correlations between miR-142-5p and HDAC5, and miR-142-5p and METTL3 in OS tissues (*p* < 0.05, Fig. 5F). Overexpression of METTL3 decreased miR-142-5p expression in OS cells and METTL3 silencing elevated miR-142-5p expression, while overexpression of HDAC5 reversed the effect of METTL3 silencing (*p* < 0.05, Fig. 5G). All these indicated that HDAC5 reduced the H3K9/K14ac level on miR-142 promoter and thereby depressed miR-142-5p expression.

### miR-142-5p targeted ARMC8

**Fig. 6:**
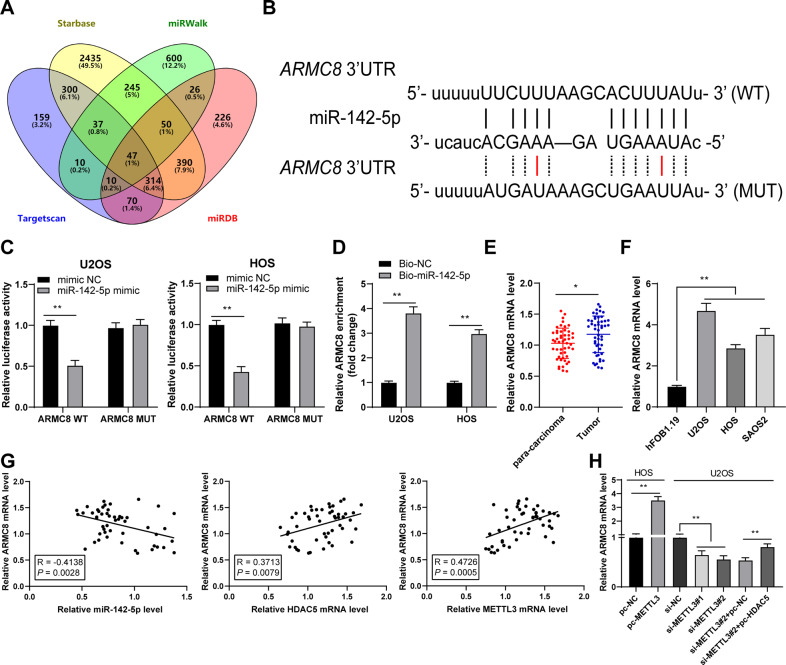
miR-142-5p targeted ARMC8. **A** The target genes of miR-142-5p were predicted through Starbase, miRWalk, Targetscan, and miRDB databases. **B** The binding site of miR-142-5p and ARMC8 in the Starbase database. **C**, **D** The binding of miR-142-5p and ARMC8 in OS cells was verified using dual-luciferase assay and RNA pull-down. **E** ARMC8 mRNA level in 50 pairs of OS tissues and adjacent tissues was determined using RT-qPCR. **F** ARMC8 mRNA level in human OS cell lines (U2OS, HOS, SAOS2) and normal osteoblasts (hFOB1.1) was determined using RT-qPCR. **G** The correlation between ARMC8, miR-142-5p, HDAC5, and METTL3 in OS tissues was analyzed using Pearson correlation analysis. **H** ARMC8 mRNA level in OS cells after intervention with METTL3 and HDAC5 expression was measured using RT-qPCR. *N* = 50. The cell experiment was repeated three times independently. Data in panels C-D/F-H are presented as mean ± standard deviation. Data in panel **E** were analyzed using paired *t*-test. Data in panels **F**/**H** were analyzed using one-way ANOVA, and data in panels **C**, **D** were analyzed using two-way ANOVA, followed by Tukey’s multiple comparisons test, *p < 0.05, **p < 0.01.

The target genes of miR-142-5p were predicted and intersected (Fig. 6A), in which ARMC8 expression is elevated in OS [24]. Therefore, we performed a dual-luciferase assay according to the obtained binding site of miR-142-5p and ARMC8 (Fig. 6B) and found that there existed a binding relationship between miR-142-5p and ARMC8 in OS cells (*p* < 0.01, Fig. 6C). RNA pull-down assay further validated the binding relationship between miR-142-5p and ARMC8 (*p* < 0.01, Fig. 6D). OS tissues and cells exhibited elevated ARMC8 expression (*p* < 0.05, Fig. 6E, F). ARMC8 expression was negatively correlated with miR-142-5p expression in OS tissues, but positively correlated with HDAC5 and METTL3 expression (*p* < 0.01, Fig. 6G). METTL3 overexpression enhanced ARMC8 mRNA expression in cells and METTL3 silencing reduced ARMC8 mRNA expression, while further HDAC5 overexpression treatment increased ARMC8 mRNA expression (*p* < 0.01, Fig. 6H). All these suggested that miR-142-5p targeted ARMC8.

### miR-142-5p silencing reversed the effect of METTL3 silencing on OS cells by upregulating ARMC8 transcription level

**Fig. 7:**
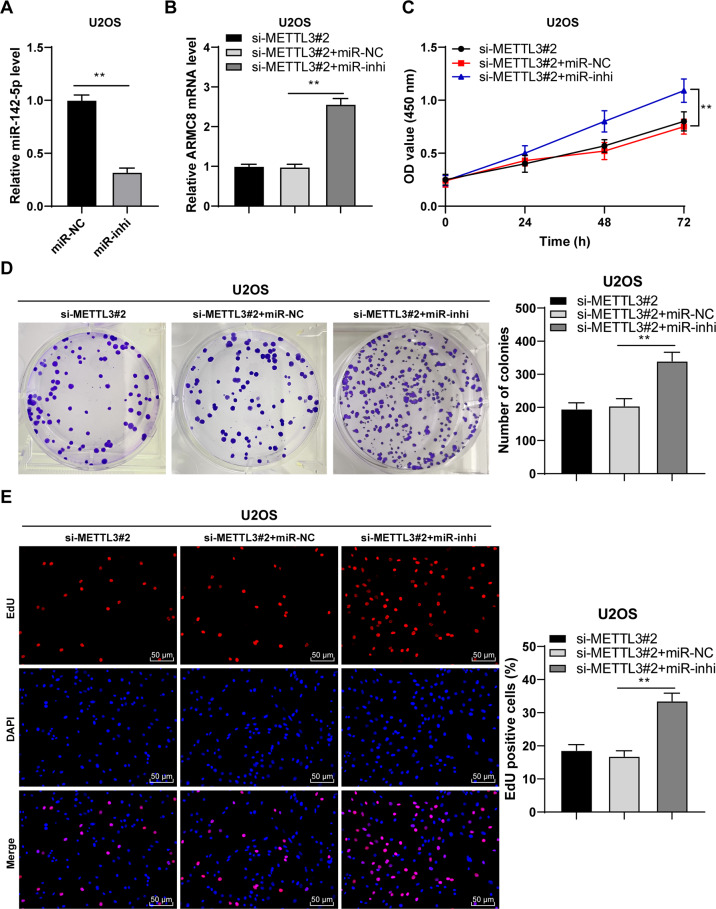
miR-142-5p silencing reversed the inhibitory effect of METTL3 silencing on OS cell proliferation by upregulating ARMC8 transcription level. miR-142-5p inhibitor (miR-inhi) was transfected into U2OS cells, with miR-NC as negative control. After 48 h, **A** miR-142-5p expression in U2OS cells was determined using RT-qPCR. Then, miR-142-5p inhibitor-transfected U2OS cells were treated with si-METTL3#2 for 48 h. **B** ARMC8 mRNA level in cells was detected using RT-qPCR. **C**–**E** The proliferation of OS cells was measured using CCK-8 assay (**C**), colony formation assay (14 days) (**D**), and EdU staining (**E**). The cell experiment was repeated three times independently. Data are presented as mean ± standard deviation. Data in panel **A** were analyzed using *t-*test. Data in panels **B**/**D**, **E** were analyzed using one-way ANOVA, and data in panel **C** were analyzed using two-way ANOVA, followed by Tukey’s multiple comparisons test, **p < 0.01.

Again, a combined experiment was conducted to verify the role of miR-142-5p/ARMC8 axis in OS cell proliferation. Transfection of miR-142-5p inhibitor (miR-inhi) successfully downregulated miR-142-5p expression in U2OS cells (*p* < 0.01, Fig. 7A). Then, miR-142-5p inhibitor-treated U2OS cells were transfected with si-METTL3#2. After miR-142-5p silencing, ARMC8 mRNA expression was increased (*p* < 0.01, Fig. 7B). The combined treatment of miR-142-5p silencing + METTL3 silencing also notably enhanced OS cell proliferation (*p* < 0.01, Fig. 7C–E). Briefly, miR-142-5p silencing attenuated the inhibition of METTL3 silencing on OS cell proliferation via upregulation of ARMC8 transcription level.

### METTL3 silencing suppressed OS proliferation in vivo via the HDAC5/miR-142-5p/ARMC8 axis

**Fig. 8:**
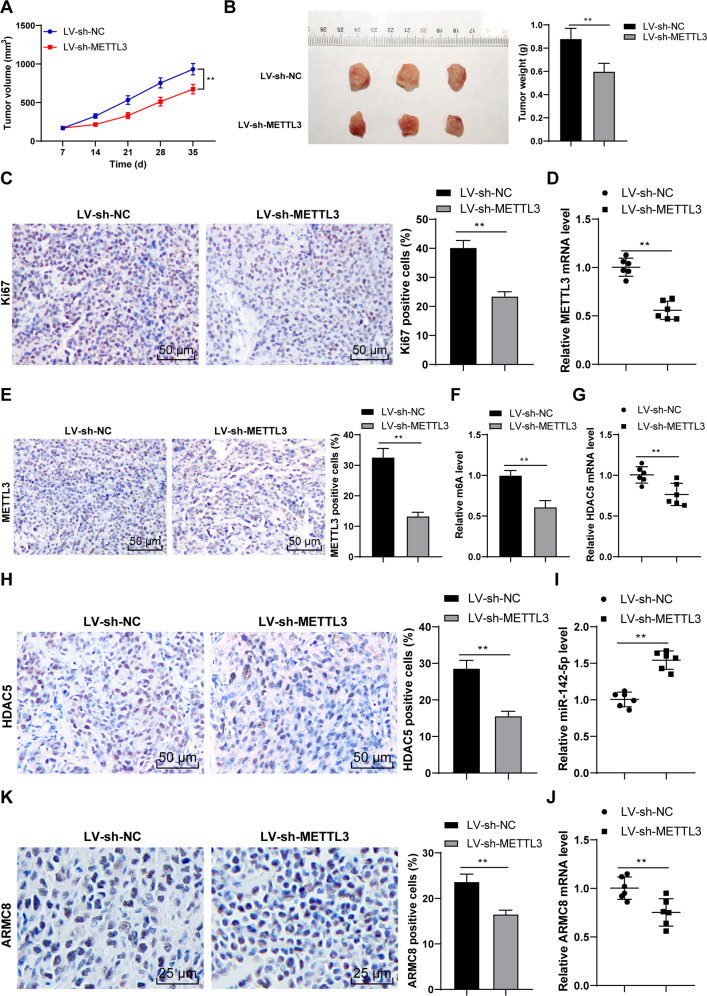
METTL3 silencing suppressed OS proliferation in vivo via the HDAC5/miR-142-5p/ARMC8 axis. The xenograft tumor model in nude mice was established with U2OS cells stably low-expressing METTL3. **A** Tumor volume recorded every week. **B** After the nude mice were euthanized on the 35th day, the tumor weight and representative images were presented. **C** The positive rate of Ki67 was detected using immunohistochemistry. **D**, **E** METTL3 level was determined using RT-qPCR and immunohistochemistry. **F** m^6^A level was quantitatively analyzed. **G**, **H** HDAC5 level was determined using RT-qPCR and immunohistochemistry. **I**, **J** miR-142-5p and ARMC8 level was determined using RT-qPCR. **K** ARMC8 level was detected using immunohistochemistry. *N* = 6. The cell experiment was repeated three times independently. Data in panels **A**–**C**/**E**, **F**/**H** are presented as mean ± standard deviation. Data in panels **B**–**K** were analyzed using *t*-test. Data in panel **A** were analyzed using two-way ANOVA, followed by Tukey’s multiple comparisons test, **p < 0.01.

Finally, we established the xenograft tumor model in nude mice to verify the role and mechanism of METTL3 in OS growth. Our results demonstrated that METTL3 silencing reduced tumor volume and weight (*p* < 0.01, Fig. 8A, B) and decreased the Ki67-positive rate (*p* < 0.01, Fig. 8C). Compared with the LV-sh-NC group, the LV-sh-METTL3 group had decreased METTL3 expression (*p* < 0.01, Fig. 8D, E) and reduced m^6^A level (*p* < 0.01, Fig. 8F). Moreover, METTL3 silencing reduced HDAC5 expression (*p* < 0.01, Fig. 8G, H), increased miR-142-5p expression (*p* < 0.01, Fig. 8I), and decreased ARMC8 mRNA expression (*p* < 0.01, Fig. 8J, K). All these indicated that METTL3 silencing suppressed OS proliferation in vivo via the HDAC5/miR-142-5p/ARMC8 axis.

## Discussion

Despite the improvement in current therapeutic strategies of OS, the overall survival of OS patients remains dismal [25]. Emerging evidence has suggested that METTL3-mediated m^6^A modification is implicated in OS tumorigenesis and development [26]. This study elucidated that METTL3-mediated m^6^A modification facilitates malignant proliferation of OS cells via the HDAC5/miR-142-5p/ARMC8 axis (Supplementary Fig. 1).

Numerous studies have revealed the promoting effect of METTL3 on OS progression, indicating the potential of METTL3 as a therapeutic target in OS [11, 12, 27]. METTL3 expression in OS tissues and cells was also much higher than that in adjacent tissues and normal osteoblasts. METTL3 is aberrantly upregulated in OS and also tightly correlated with the metastasis and prognosis of OS patients [9, 28]. Consistently, our results demonstrated that METTL3 expression was correlated with tumor size, clinical stage, and distance metastasis, and the overall survival rate was worse in patients with higher METTL3 expression. To probe into the specific effect of METTL3 on OS cell proliferation, we intervened in METTL3 expression. Our results exhibited that METTL3 silencing notably reduced OS cell proliferation, while METTL3 overexpression enhanced OS cell proliferation. Knockdown of METTL3 has been validated to depress OS cell growth [12]. Briefly, METTL3 was highly expressed in OS, and METTL3 silencing suppressed malignant proliferation of OS cells.

Thereafter, we shifted to exploring the downstream mechanism of METTL3 in OS cell proliferation. METTL3-mediated m^6^A modification is implicated in diverse biological processes including tumorigenesis, and m^6^A level and METTL3 expression are notably elevated in OS cells [11]. Silencing METTL3 can downregulate m^6^A methylation level in OS cells [12]. We also found that the OS tissues and cells had increased m^6^A level, while METTL3 silencing decreased m^6^A level. METTL3 overexpression has been documented to enhance HDAC5 expression through m^6^A modification, suggesting that HDAC5 is a crucial target of METTL3 [14]. Importantly, aberrantly elevated HDAC5 expression has been observed in OS tissues [16]. HDAC5 is recruited to the long telomeres of OS cells, and HDAC5 depletion is essential for the sensitivity of OS cells to chemotherapy [29]. Similarly, our results exhibited that OS tissues and cells had elevated HDAC5 expression. METTL3 recognizes m^6^A-modified RNAs and controls diverse processes of RNAs, such as RNA stability, translation, or splicing [30]. Our results exhibited that METTL3 silencing notably reduced m^6^A modification level on HDAC5 mRNA, weakened HDAC5 mRNA stability, and suppressed HDAC5 expression. Then, we transfected pc-HDAC5 into si-METTL3-treated U2OS cells. HDAC5 overexpression accelerates OS cell proliferation by upregulating the oncogene Twist 1 [16]. Consistently, our findings demonstrated that HDAC5 overexpression dramatically enhanced OS cell proliferation, indicating that HDAC5 overexpression partially reversed the inhibitory effect of METTL3 silencing on OS cell proliferation.

HDAC5 exhibits histone deacetylase activity, and loss of HDAC5 elevates the acetylation level of histones H3/H4 [21]. Accordingly, we observed the reduced H3/H4ac level in OS cells. It has been reported that HDAC5 can enhance H3K27 methylation and reduce H3K9/K14 acetylation, thus repressing miR-142-5p expression [22]. Our results exhibited that HDAC5 and H3K9/K14ac were enriched on miR-142 promoter in OS cells. Overexpression of METTL3 enhanced the enrichment of HDAC5 on miR-142 promoter and reduced the enrichment of H3K9/K14ac, while METTL3 silencing led to the opposite trends; after overexpression of HDAC5, the enrichment of HDAC5 on miR-142 promoter was enhanced and the enrichment of H3K9/K14ac was reduced. Particularly, miR-142-5p is correlated with the pathogenesis of OS and accepted as a critical indicator of metastasis and therapeutic response [25]. Consistent with the findings in previous study [23], we presented the declined miR-142-5p expression in OS tissues and cells, and the negative correlations between miR-142-5p and HDAC5, and miR-142-5p and METTL3. Briefly, HDAC5 reduced the H3K9/K14ac level on miR-142 promoter and thereby depressed miR-142-5p expression. Furthermore, the target genes of miR-142-5p were predicted, among which we focused on miR-142-5p. The binding relationship between miR-142-5p and ARMC8 was validated in OS cells. A prior study has illustrated that ARMC8 functions as an oncogenic factor in OS [24]. OS tissues and cells also presented enhanced ARMC8 expression, and ARMC8 was negatively correlated with miR-142-5p in OS tissues, but positively correlated with HDAC5 and METTL3 expressions. To validate the role of miR-142-5p/ARMC8 in OS cell proliferation, we transfected miR-142-5p inhibitor into si-METTL3-treated U2OS cells. Our results exhibited that miR-142-5p silencing enhanced ARMC8 transcription level and facilitated OS cell proliferation. miR-142-5p silencing expedites the malignant progression of OS, while miR-142-5p overexpression depresses proliferation and facilitates OS apoptosis [23]. ARMC8 overexpression induces epithelial-mesenchymal transition (EMT), consequently triggering OS cell migration and invasion [24]. Briefly, miR-142-5p silencing reversed the inhibitory effect of METTL3 silencing on OS cell proliferation by upregulating ARMC8 transcription level. Finally, we established the xenograft tumor model in nude mice to verify the role and mechanism of METTL3 in OS growth. Our results verified that METTL3 silencing suppressed OS proliferation in vivo via the HDAC5/miR-142-5p/ARMC8 axis.

To sum up, METTL3-mediated m^6^A modification upregulated HDAC5 expression, thereby reducing the enrichment of H3K9/K14ac on miR-142 promoter, suppressing miR-142-5p expression, enhancing ARMC8 transcription level, and eventually facilitating OS cell proliferation. This study merely determined the effect of METTL3 on HDAC5, but whether there were other genes regulated by METTL3 remained unknown. Similarly, the role of other downstream genes of HDAC5 and miR-142-5p needed to be explored. In addition, this study just explored the impact of ARMC8 mRNA level on OS cells, but the effect of ARMC8 protein level in in vitro experiments is unclear. Finally, whether METTL3 also affected other functions of OS cells, such as migration and EMT, remains further exploration. In the future, we will validate the effect of METTL3 on other functions of OS cells and investigate other potential mechanisms of HDAC5 to confer novel insights for the management of OS.

## Materials and methods

### Clinical data

OS tissues and adjacent non-OS tissues were obtained from 50 OS patients (26 males and 24 females) and then stored at −80 °C. All OS patients did not receive chemotherapy or radiotherapy before.

### Cell culture

Human OS cell lines U2OS (derived from sampling site: bone; tibia), HOS (derived from sampling site: bone; right femur), and SAOS2 (derived from sampling site: bone) and normal osteoblast hFOB1.19 (derived from sampling site: fetal bone; limb) were supplied by Tongpai Biotechnology Co., Ltd (Shanghai, China). All cells were identified by short tandem repeat (STR) and cultured in RPMI 1640 medium (Gibco, Rockville, MD, USA) containing 10% fetal bovine serum (FBS) at 37 °C with 5% CO_2_.

### Cell treatment

pcDNA 3.1 METTL3, pcDNA 3.1 HDAC5, METTL3 small interfering RNA (siRNA), HDAC5 siRNA, and their negative controls (NCs) were obtained from RiboBio (Guangzhou, China). miR-142-5p mimic or mimic NC, and miR-142-5p inhibitor or inhibitor NC were supplied by GenePharma (Shanghai, China). For cell transfection, U2OS or HOS cells were seeded into 24-well plates (2 × 10^5^ cell/well). After 12 h, these cells were transfected using Lipofectamine 3000 reagent (Invitrogen, Carlsbad, CA, USA). The cells were harvested after 48 h for subsequent experimentation.

### Cell counting kit-8 (CCK-8) assay

U2OS or HOS cells were transferred into 96-well plates (2 × 10^3^ cells/well). After 24 h, these cells were subjected to incubation with 10 μL CCK-8 reagent (Beyotime, Shanghai, China) for 4 h. The absorbance at 450 nm was determined using a microplate reader (Bio-Rad, Philadelphia, PA, USA). Similarly, the absorbance of cells was measured after 48 h and 72 h of incubation. Finally, the proliferation curve of cells in each group was drawn.

### Colony formation assay

After transfection, U2OS or HOS cells were seeded into 12-well plates (1000 cells/well) and maintained in RPMI 1640 medium (containing 10% FBS) at 37 °C for 14 d. Afterward, the cells were rinsed with phosphate-buffered saline (PBS), treated with 4% paraformaldehyde (PFA) (Sigma–Aldrich, Merck KGaA, Darmstadt, Germany) for 20 min, and stained with 0.1% crystal violet (Solarbio, Beijing, China) for 15 min. Finally, the cell colonies were observed and counted under a light microscope (Olympus, Tokyo, Japan).

### 5-ethynyl-2’-deoxyuridine (EdU) staining

After transfection, U2OS or HOS cells (1 × 10^4^) were maintained in RPMI 1640 medium containing EdU (50 μM, RiboBio) at 37 °C for 4 h, treated with 4% PFA for 30 min, and maintained with glycine for 10 min. Then, these cells were treated with 0.3% Triton-X-100 solution for 15 min and rinsed with PBS three times. Next, each well was treated with 200 μL 1× Apollo reaction liquid for 20 min and the nuclei were stained with 4’,6-diamidino-2-phenylindole (5 μg/mL). The cells were imaged by a fluorescence microscope (Olympus) and the number of EdU-positive cells was calculated.

### Bioinformatics analysis

The downstream genes of miR-106a-5p were predicted through Starbase database (http://starbase.sysu.edu.cn/index.php) [31], Targetscan database (http://www.targetscan.org/vert_71/) [32], miRWalk database (http://mirwalk.umm.uni-heidelberg.de/) [33].

### Quantification of m^6^A RNA methylation level

TRIzol reagent (Invitrogen) was adopted for the extraction of total RNA. The m^6^A RNA methylation quantification kit (ab185912, Abcam Inc., Cambridge, MA, USA) was employed for m^6^A quantification. Briefly, the relevant solution was added to a well containing 200 ng RNA for m^6^A RNA capture. Then, 100 μL termination solution was added to terminate enzyme reaction. Finally, the absorbance at 450 nm was determined using a microplate reader to measure the m^6^A level.

### m^6^A methylated RNA immunoprecipitation-qPCR (MeRIP-qPCR)

MeRIP-qPCR was performed using the MeRIP kit (Merck Millipore, Billerica, MA, USA) as described in the previous literature [11]. Shortly, the total RNA of U2OS or HOS cells was extracted using TRIzol reagent. Then, RNA was incubated with anti-m^6^A (ab208577, Abcam) or anti-IgG (ab170190, Abcam) and protein A/G magnetic beads in IP buffer overnight. After ethanol precipitation, the immunoprecipitated m^6^A RNA was analyzed using reverse transcription quantitative polymerase chain reaction (RT-qPCR).

### RNA stability detection

U2OS cells were subjected to actinomycin D (5 μg/mL) treatment for 0, 2, 4, and 6 h. Afterward, the cells were collected and RNA was extracted for RT-qPCR to detected HDAC5 level.

### Chromatin immunoprecipitation (ChIP)-qPCR

ChIP was performed using the Magna ChIP Assay kit (Millipore) as described in the previous literature [22]. Briefly, U2OS or HOS cells were fixed with 1% PFA for 10 min and harvested using ChIP lysis buffer. Subsequently, the DNA was sonicated and the supernatant was collected for incubation with anti-HDAC5 (98329 S, CST, Beverly, MA, USA), anti-H3K9ac (ab32129, Abcam), and H3K14ac (ab52946, Abcam) at 4 °C overnight, with IgG (ab205718, Abcam) as NC. The immune complex was precipitated with protein A agarose beads, washed, and then eluted and purified using 100 μL TE buffer containing 0.5% SDS and 200 μg/mL protease K. ChIP-derived DNA was quantified using RT-qPCR. miR-142 promoter primers were as follows: forward primer 5’-GAAAGGCCTCCATGGCTTTCCT-3’ and reverse primer 5’-CGGCTGCATCAGGGTTCC -3’.

### Dual-luciferase assay

The sequence of armadillo-repeat-containing protein 8 (ARMC8) containing wild-type miR-142-5p binding site was cloned into pGL3 reporter gene vector (Promega, Madison, Wisconsin, USA) to construct wild-type ARMC8. Meanwhile, the sequence of ARMC8 containing mutant-type miR-142-5p binding site was cloned into pGL3 to construct mutant-type ARMC8. Then, miR-142-5p mimic or mimic NC was co-transfected with the constructed reporter plasmid into U2OS or HOS cells. After 48 h incubation, the culture medium was discarded and cells were harvested. The harvested cells were lysed to produce cell lysate. Luciferase activity was measured using the Dual-Glo® luciferase analysis system (Promega).

### RNA pull-down

Biotin-labeled miR-142-5p (Bio-miR-142-5p) or miR-NC (Bio-NC) supplied by RiboBio was transfected into U2OS or HOS cells (3 × 10^5^). After 48 h, these cells were lysed, followed by 2 h incubation with streptavidin beads (Thermo Fisher Scientific Inc., Waltham, MA, USA). The beads were then collected and rinsed with PBS twice. Subsequently, RNA attached to beads was eluted and washed using the RNeasy minikit (Qiagen, Hilden, Germany). Finally, RT-qPCR was performed to evaluate the abundance of ARMC8.

### Establishment of the xenograft tumor model in nude mice

Female BALB/c mice (5 week old; 15–18 g) obtained from Beijing Vital River Laboratory Animal Technology Co., Ltd [SYXK (Beijing) 2017-0033] were kept under a 12 h light/dark cycle at 22 ± 2 °C with 45–55% humidity. Each mouse had free access to food and water. Firstly, U2OS cells were infected with lentivirus vector with low expression of METTL3. U2OS cells with stable METTL3 expression were screened using puromycin for subsequent experiments. U2OS cells (1 × 10^6^) were injected subcutaneously to the right side of each mouse (the mice were numbered according to their weight, and the experimenters randomly divided the nude mice into 12 groups by the random number method, with 12 mice in each group). The status of mice was detected every 2 days. Tumor growth and volume were measured once a week. Tumor volume was measured: Volume = Width^2^ × Length/2. On the 35th day after the operation, nude mice were euthanized by intraperitoneal injection of ≥100 mg/kg pentobarbital sodium. Tumor resection was performed and tumor weight was recorded. Weight loss >10% of the body weight or the maximum diameter of the tumor >1.5 cm was the humane end point. During the experiment, great efforts were made to reduce the pain and depression of mice, and there was no midway death of animals. On the one hand, considering animal ethics, we reduced the number of mice used as much as possible. On the other hand, to ensure the reliability of experimental data and sample size, we collected tumor specimens from six mice for immunohistochemical staining, and the tumor specimens of remaining six mice were subjected to RT-qPCR.

### Immunohistochemistry

The resected tumor was fixed with 4% PFA, paraffin-embedded, and sectioned (4 μm). After dewaxing and rehydration, the sections were treated with H_2_O_2_ for 8 min to reduce endogenous peroxidase activity and blocked with 3% bovine serum albumin for 1 h to avoid specific reaction. Then, the sections were incubated with anti-Ki67 (ab15580, Abcam) or METTL3 (ab195352, Abcam), ARMC8 (ab272621, Abcam), and HDAC5 (ab55403, Abcam) overnight. Afterward, the sections were subjected to incubation with the secondary antibody (ab205718, Abcam) for 30 min, developed using 2,4-diaminobutyric acid (Yeasen Biotech, Shanghai, China), and counterstained using hematoxylin (Beyotime). After dehydration, the sections were sealed with neutral balsam and observed under a microscope (Olympus CKX51) by a double-blind method. Five high-power microscope fields were randomly selected for each section, and the number of positive cells in each field was counted [34].

### RT-qPCR

The total RNA was extracted from cells or tissues using TRIzol reagent (Invitrogen) and RNA concentration was determined using the NanoDrop instrument. The RNA of A260/280 at 1.8–2.0 was selected for subsequent experiments. RNA was reverse transcribed into cDNA using the PrimeScript RT kit (Takara, Dalian, China). RT-qPCR was performed using the SYBR Green system (Takara) on the ABI 7500 Fast instrument. Supplementary Table 1 shows the PCR primers. The relative expression of genes was calculated using the 2^-ΔΔCt^ method [35], with GAPDH and U6 as internal reference [36].

### Western blot

Briefly, total protein of cells or tissues was extracted using radio-immunoprecipitation assay buffer containing 1% protease inhibitor, and the protein concentration was examined using bicinchoninic acid kit (Beyotime). Subsequently, the protein was separated by 10% SDS-PAGE and transferred onto PVDF membranes (Millipore). Afterward, the membranes were blocked with 5% skim milk at 37 °C for 2 h and subjected to incubation with the primary antibodies at 4 °C overnight: METTL3 (ab195352, 1:1000, Abcam), HDAC5 (ab55403, 1:500, Abcam), H3ac (PA5-114693, 1:500, Invitrogen), H4ac (PA5-32029, 1:5000, Invitrogen), and β-actin (ab8227, 1:1000, Abcam). After tris-buffered saline-tween (TBST) buffer rinsing, the membranes were subjected to incubation with the secondary antibody (ab6721, 1:2000, Abcam) at 37 °C for 1 h and rinsed with PBS three times. The protein bands were examined using enhanced chemiluminescence (Beyotime). The gray value was analyzed using Image J (National Institutes of Health, Bethesda, Maryland, USA).

### Statistical analysis

Data analysis and map plotting were performed using the SPSS 21.0 (IBM Corp., Armonk, NY, USA) and GraphPad Prism 8.0 (GraphPad Software Inc., San Diego, CA, USA). The data complied with the assumption of normality and homogeneity of variance. The measurement data are presented as mean ± standard deviation. The *t-*test was utilized for comparisons between two groups. One-way or two-way analysis of variance (ANOVA) was adopted for comparisons among multiple groups, followed by Tukey’s multiple comparison test. The enumeration counting data were presented as cases. The Chi-square test was utilized for comparisons between groups. Kaplan–Meier survival curve and Log-rank test were adopted to determine the correlation between METTL3 expression and the prognosis of OS patients. Pearson correlation analysis was utilized to determine the correlation between the factors. All *p*-values were two sided and a value of *p* < 0.05 meant statistically significant.

## Supplementary information

Supplementary Table 1

Supplementary Table 2

Supplementary Figure Legends

Supplementary Figure 1

Western blots

## Data Availability

The datasets used and analyzed during the current study are available from the corresponding author on reasonable request.
