# Supplementary material for: METTL3 stabilizes HDAC5 mRNA in an m6A-dependent manner to facilitate malignant proliferation of osteosarcoma cells
Source: Cell Death Discov. 2022 Apr 8;8:179. doi: 10.1038/s41420-022-00926-5 (PMC8993827; doi:10.1038/s41420-022-00926-5)
Supplement: Supplementary file 1 — Supplementary Table 1 [file 41420_2022_926_MOESM1_ESM.docx]

| Name | Sequence (5’-3’) |
| --- | --- |
| METTL3 | F: TGGGGGTATGAACGGGTAGA |
|  | R: TGGTTGAAGCCTTGGGGATT |
| HDAC5 | F: TGTGACAGTGGAGGTGAAGC |
|  | R: TCCACAGAGCCCACCAGAG |
| miR-142-5p | F: GCCGGGCACATAAAGTAGAAAG |
|  | R: CTGGTGTCGTGGAGTCGGCAA |
| ARMC8 | F: ATCCGCATGAGCGTCCTTTC |
|  | R: CTTTCTGGGGATCAGGGTCA |
| GAPDH | F: ATGGTTTACATGTTCCAATATGA |
|  | R: TTACTCCTTGGAGGCCATGTGG |
| U6 | F: TCGCTTCGGCAGCACATATACT |
|  | R: GCTTCACGAATTTGCGTGTCATC |

**Supplementary Table 1** PCR primer sequence

**Note:** METTL3: methyltransferase-like 3; HDAC5: histone deacetylase 5; miR-142-5p: microRNA-142-5p; ARMC8: armadillo repeat-containing protein 8; GAPDH: glyceraldehyde-3-phosphate dehydrogenase
