# Supplementary material for: METTL3 stabilizes HDAC5 mRNA in an m6A-dependent manner to facilitate malignant proliferation of osteosarcoma cells
Source: Cell Death Discov. 2022 Apr 8;8:179. doi: 10.1038/s41420-022-00926-5 (PMC8993827; doi:10.1038/s41420-022-00926-5)
Supplement: Supplementary file 2 — Supplementary Table 2 [file 41420_2022_926_MOESM2_ESM.docx]

**Supplementary Table 2** Correlation between METTL3 mRNA and clinicopathological characteristics of osteosarcoma patients

| Characteristic | Number | METTL3 mRNA | | *p* value |
| --- | --- | --- | --- | --- |
|  | (N = 50) | Low expression  (N = 25) | High expression  (N = 25) |  |
| Age |  | | | 0.777 |
| < 18 | 23 | 11 | 12 |  |
| ≥ 18 | 27 | 14 | 13 |  |
| Gender |  | | | 0.571 |
| Female | 26 | 12 | 14 |  |
| Male | 24 | 13 | 11 |  |
| Tumor size |  | | | **0.024** |
| < 5 cm | 26 | 17 | 9 |  |
| ≥ 5 cm | 24 | 8 | 16 |  |
| Anatomic location |  |  |  | 0.396 |
| Femur/tibia | 25 | 14 | 11 |  |
| Elsewhere | 25 | 11 | 14 |  |
| Clinical stage |  |  |  | **0.005** |
| Ⅰ/ⅡA | 26 | 18 | 8 |  |
| ⅠB/Ⅲ | 24 | 7 | 17 |  |
| Distant metastasis |  |  |  | **0.011** |
| No | 23 | 16 | 7 |  |
| Yes | 27 | 9 | 18 |  |

**Note:** Chi square test was used for data comparison.
