# Supplementary material for: METTL3 stabilizes HDAC5 mRNA in an m6A-dependent manner to facilitate malignant proliferation of osteosarcoma cells
Source: Cell Death Discov. 2022 Apr 8;8:179. doi: 10.1038/s41420-022-00926-5 (PMC8993827; doi:10.1038/s41420-022-00926-5)
Supplement: Supplementary file 3 — Supplementary Figure Legends [file 41420_2022_926_MOESM3_ESM.docx]

**Supplementary Fig. 1 Mechanism of METTL3 in regulating malignant proliferation of OS.** METTL3-mediated m^6^A modification upregulated HDAC5 expression in OS cells; HDAC5 act on miR-142 promoter, reduced the enrichment of H3K9/K14ac on its promoter, inhibit the expression of miR-142-5p, thereby suppressing the binding of miR-142-5p and ARMC8, upregulating ARMC8 level, and eventually promoting the malignant proliferation of OS cells.
