# Supplementary figures and images for: METTL3 stabilizes HDAC5 mRNA in an m6A-dependent manner to facilitate malignant proliferation of osteosarcoma cells
Source: Cell Death Discov. 2022 Apr 8;8:179. doi: 10.1038/s41420-022-00926-5 (PMC8993827; doi:10.1038/s41420-022-00926-5)

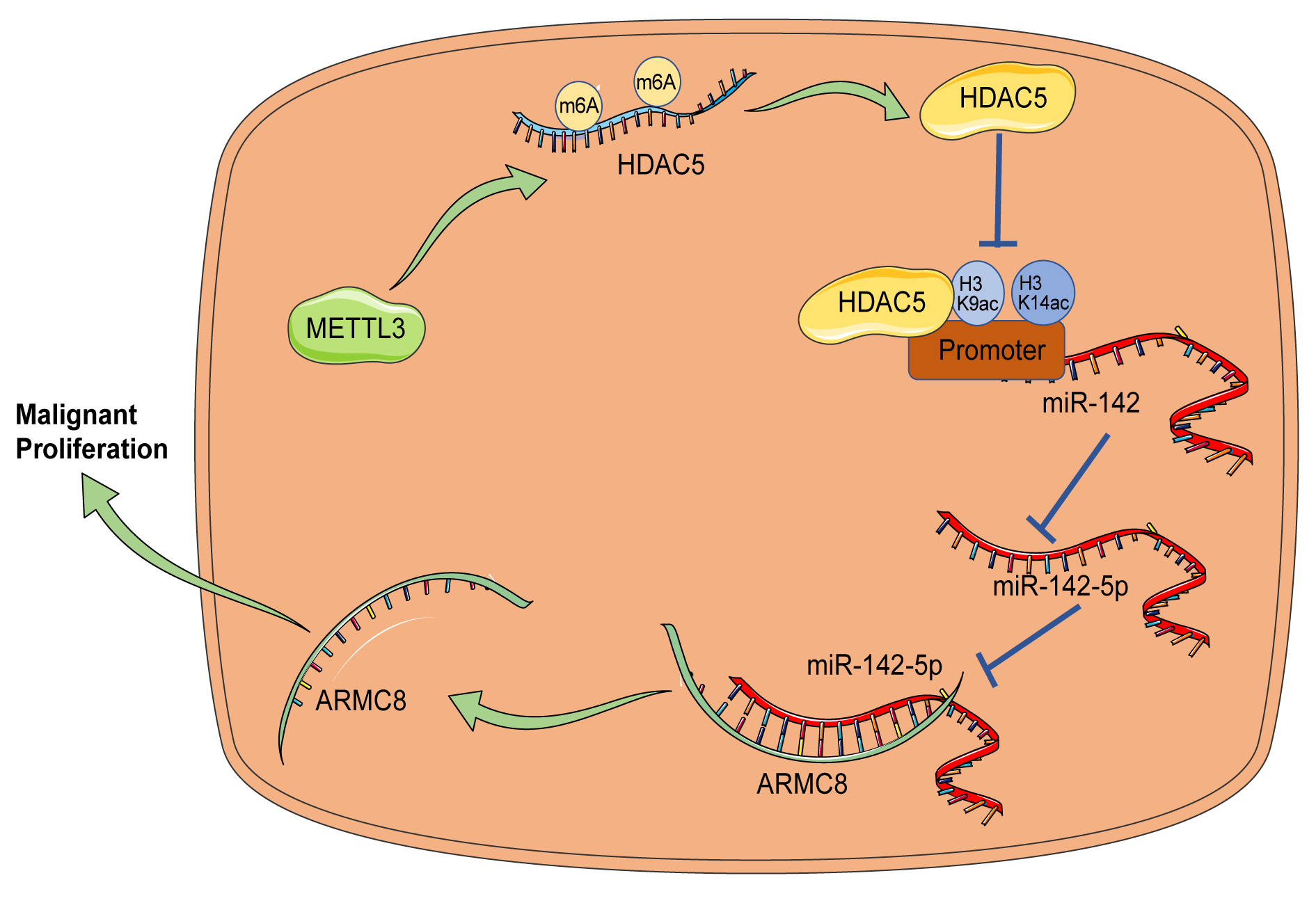

Supplement: Supplementary file 4 — Supplementary Figure 1 [file 41420_2022_926_MOESM4_ESM.tif]
