# Supplementary material for: METTL3 stabilizes HDAC5 mRNA in an m6A-dependent manner to facilitate malignant proliferation of osteosarcoma cells
Source: Cell Death Discov. 2022 Apr 8;8:179. doi: 10.1038/s41420-022-00926-5 (PMC8993827; doi:10.1038/s41420-022-00926-5)

**Figure 1B**

**METTL3**


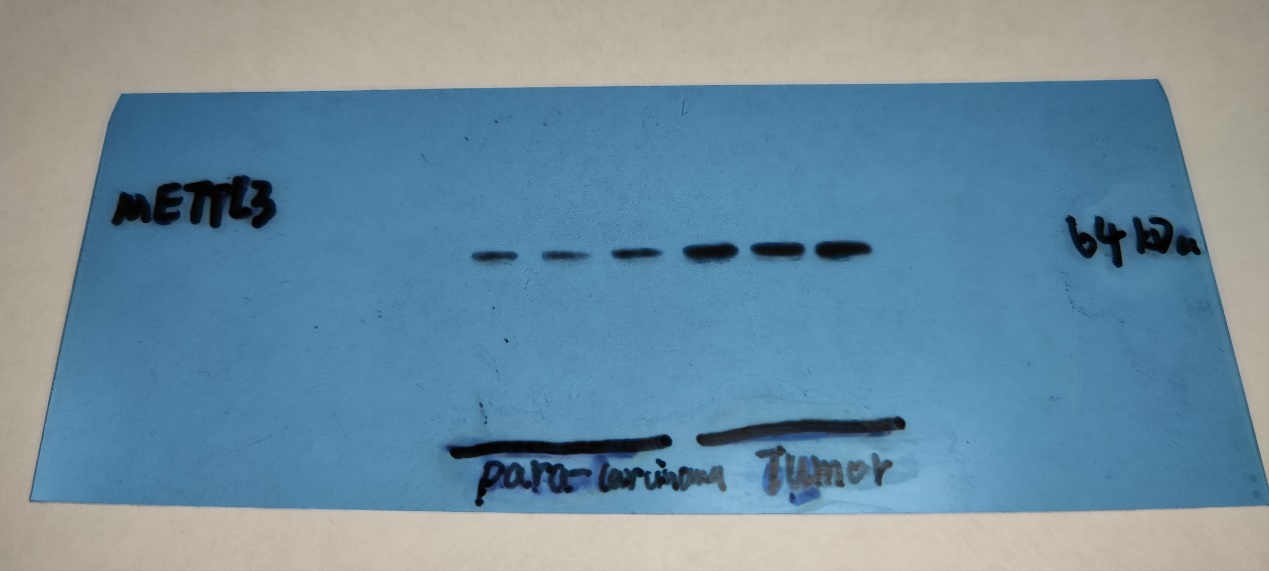


**Figure 1B**

**β-actin**


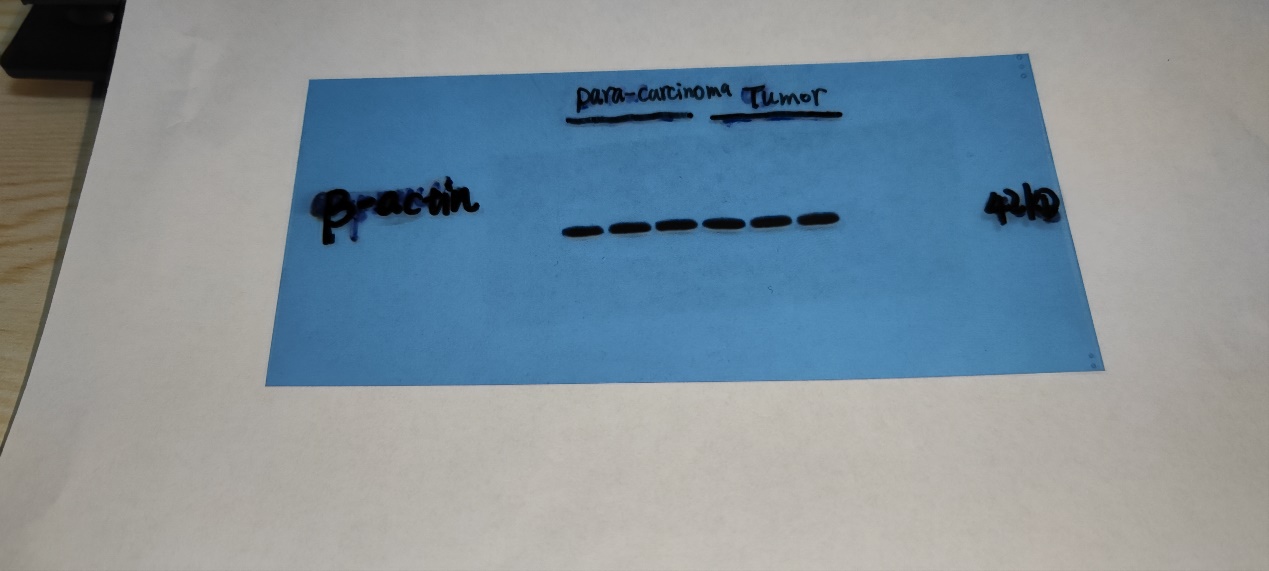


**Figure 1D**

**METTL3**


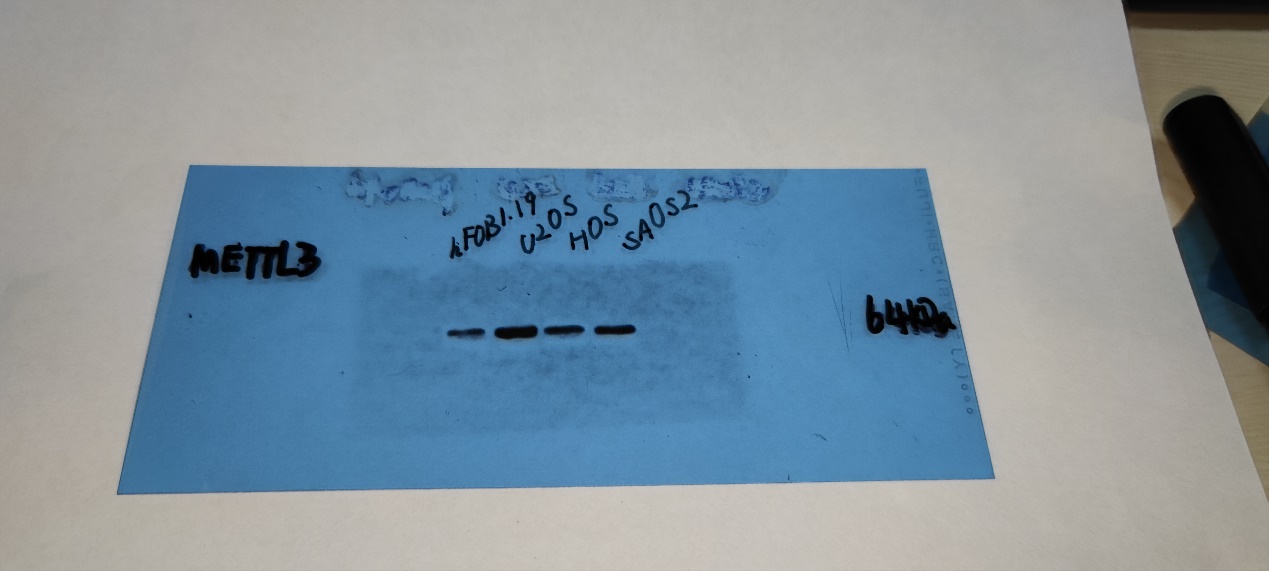


**Figure 1D**

**β-actin**


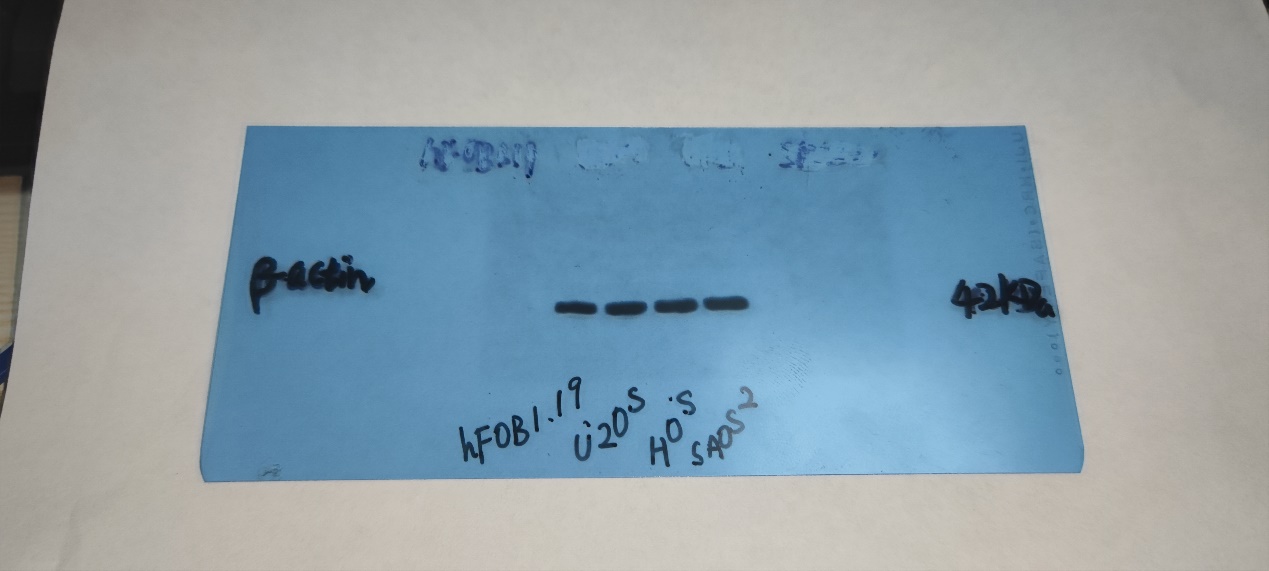


**Figure 2B**

**METTL3**


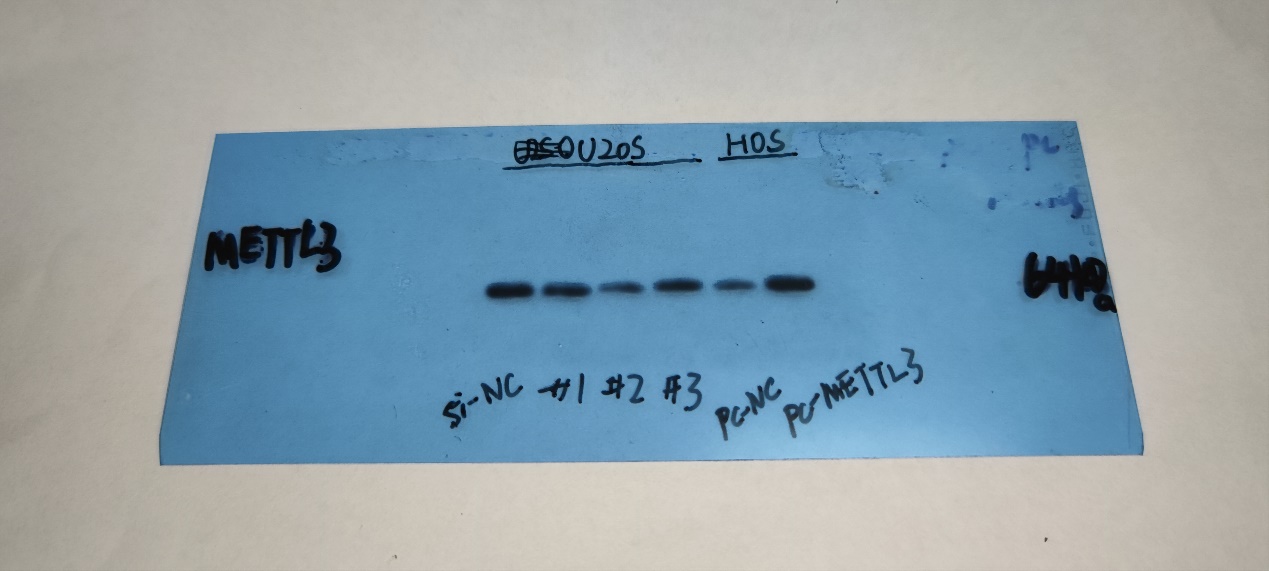


**Figure 2B**

**β-actin**


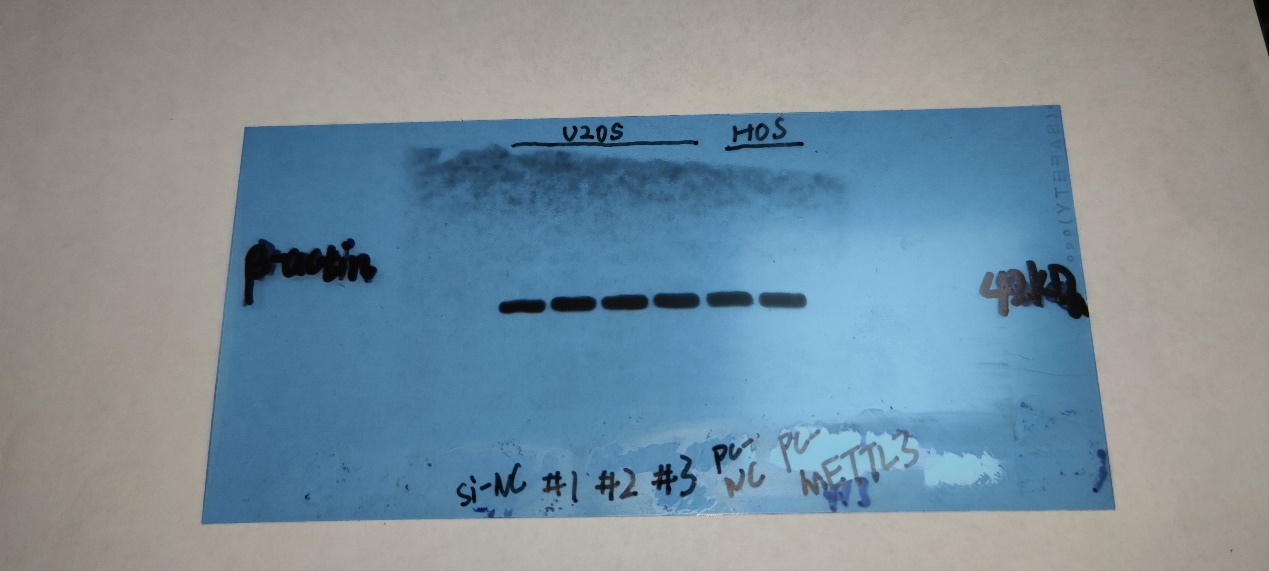


**Figure 3D**

**HDAC5**


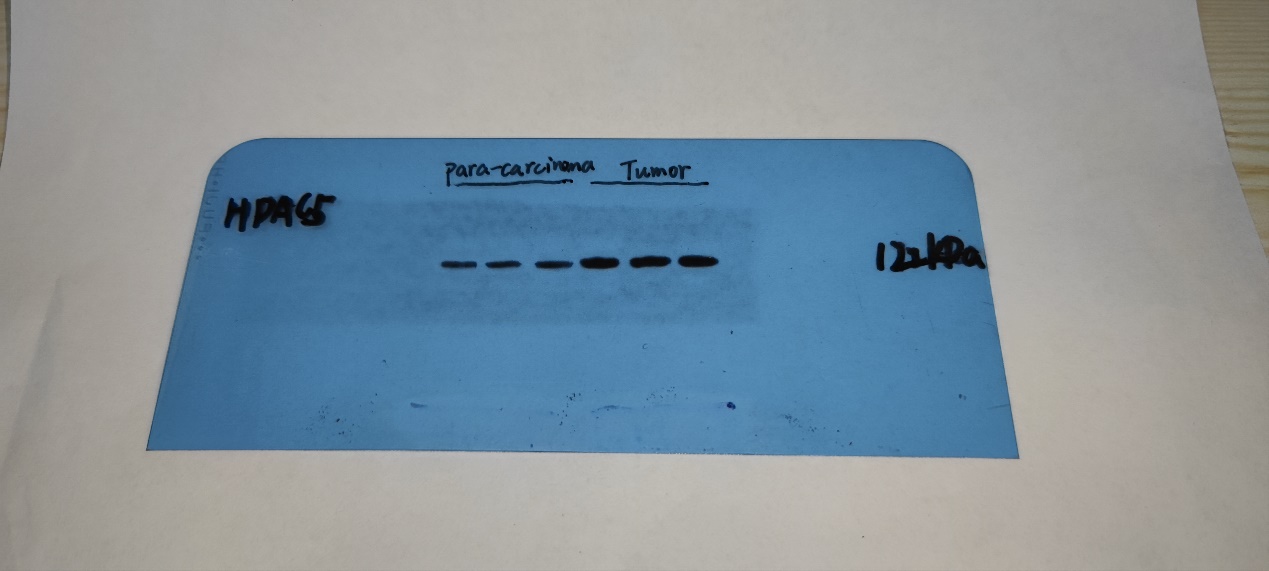


**Figure 3D**

**β-actin**


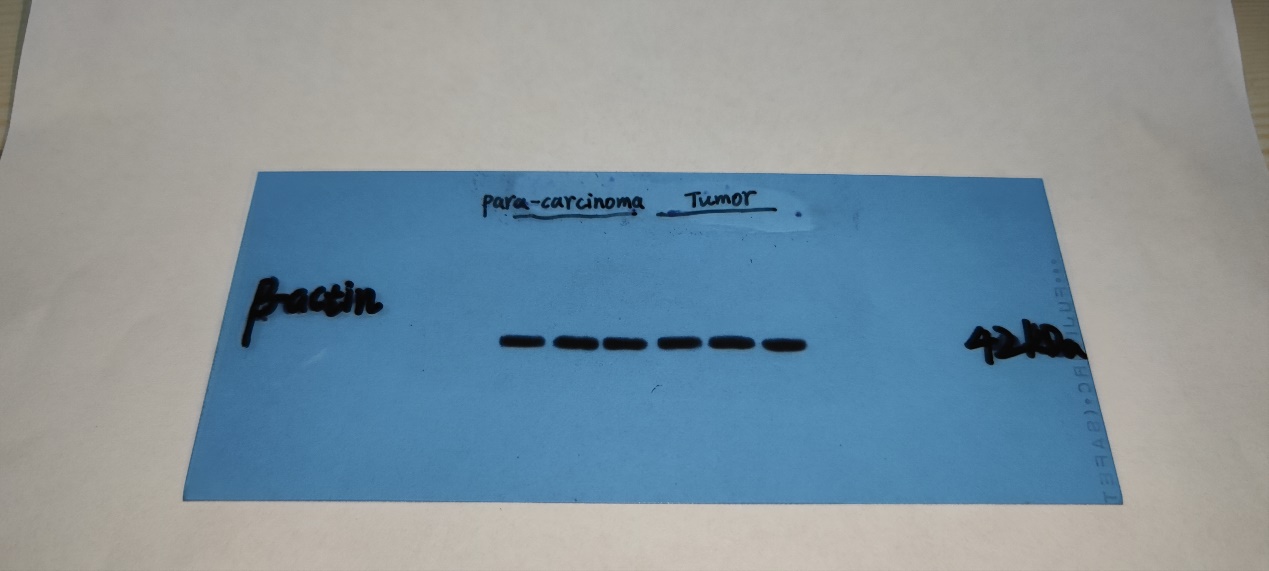


**Figure 3F**

**HDAC5**


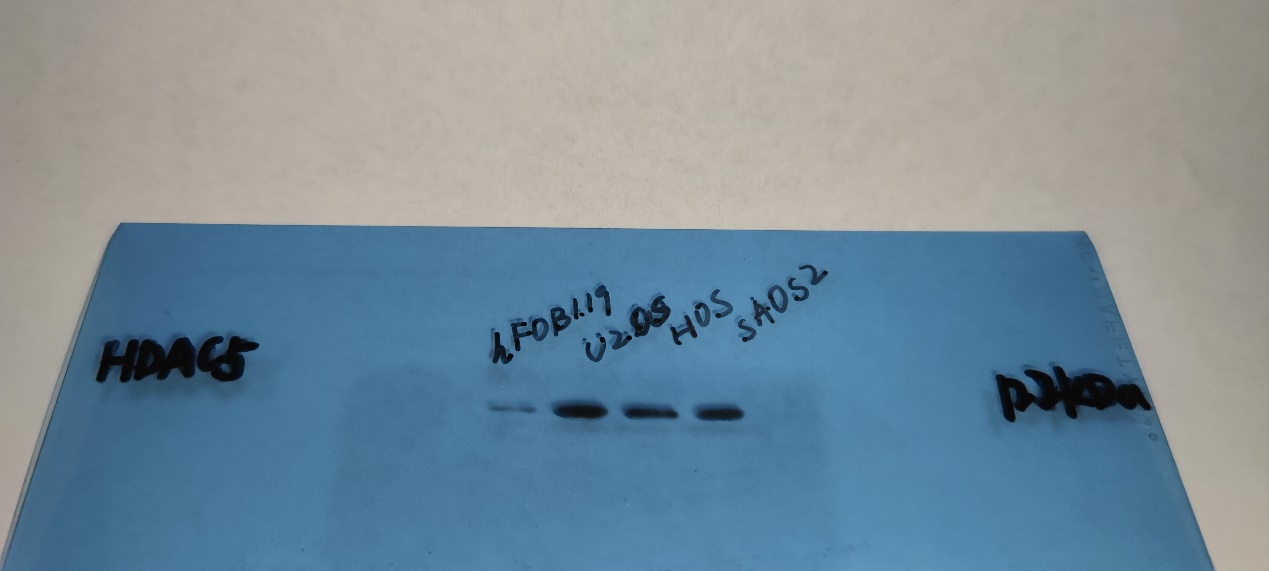


**Figure 3F**

**β-actin**


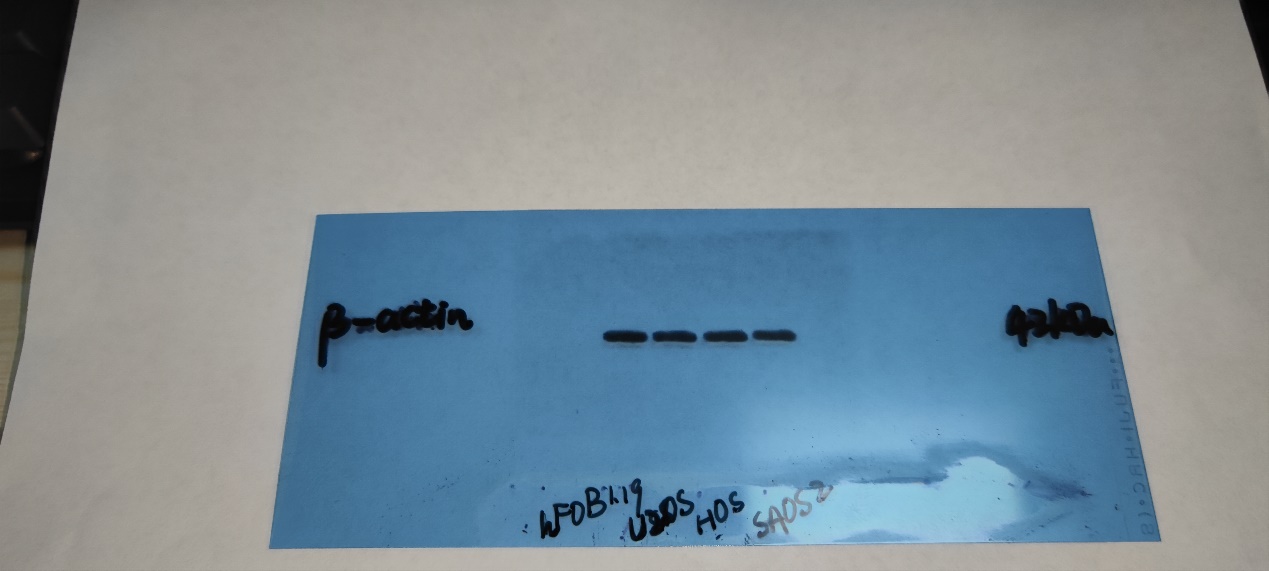


**Figure 3J**

**HDAC5**


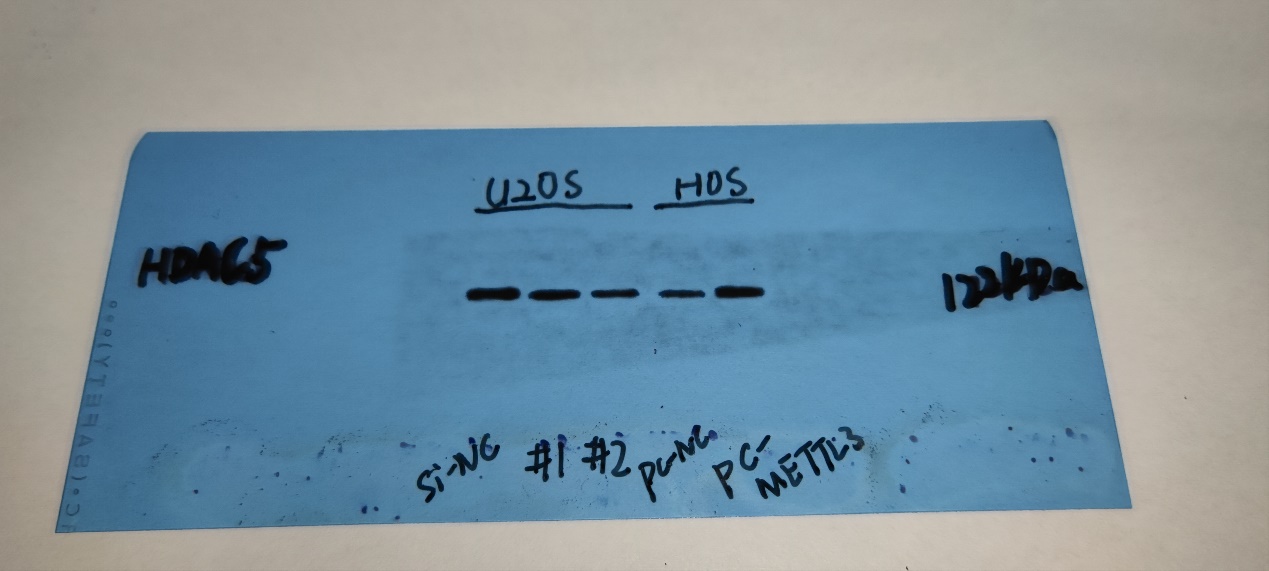


**Figure 3J**

**β-actin**


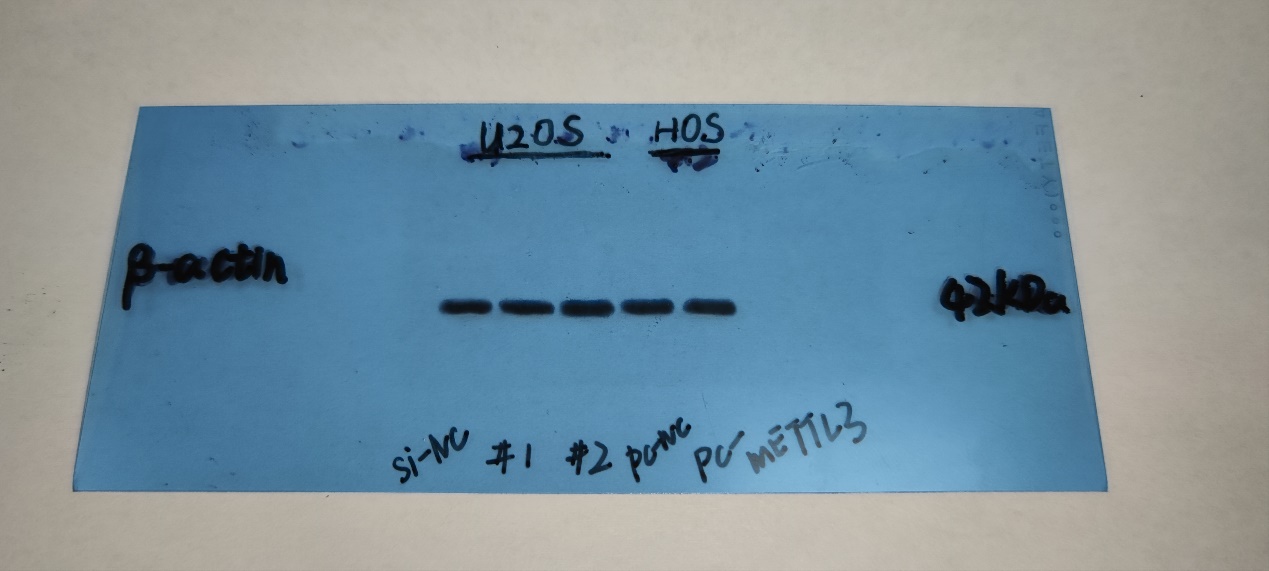


**Figure 4B**

**HDAC5**


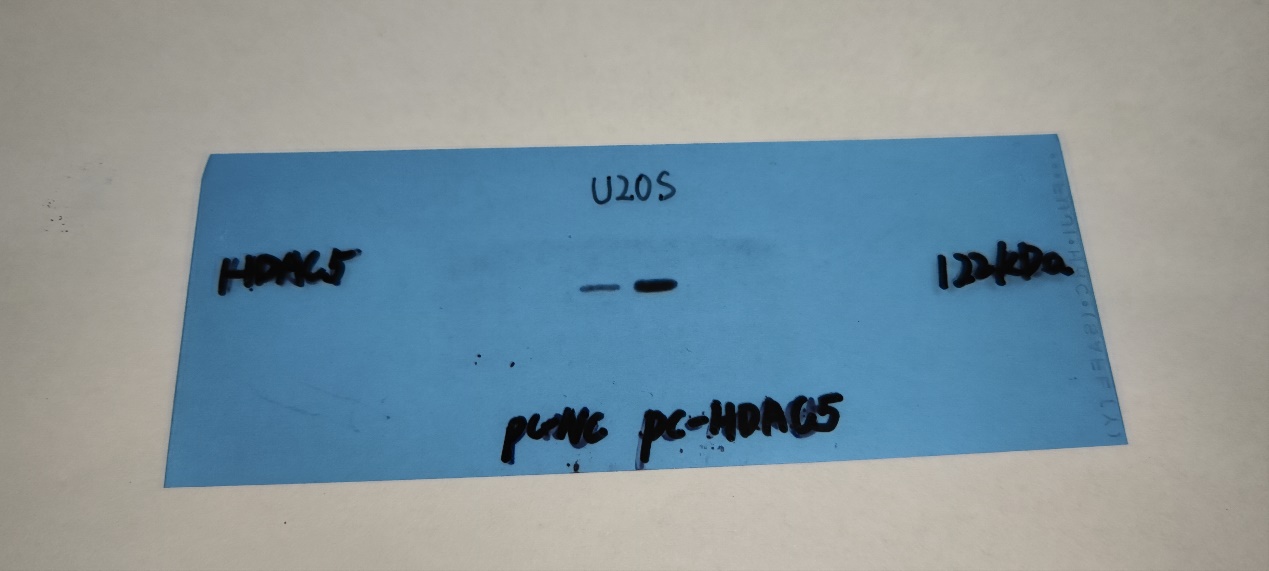


**Figure 4B**

**β-actin**


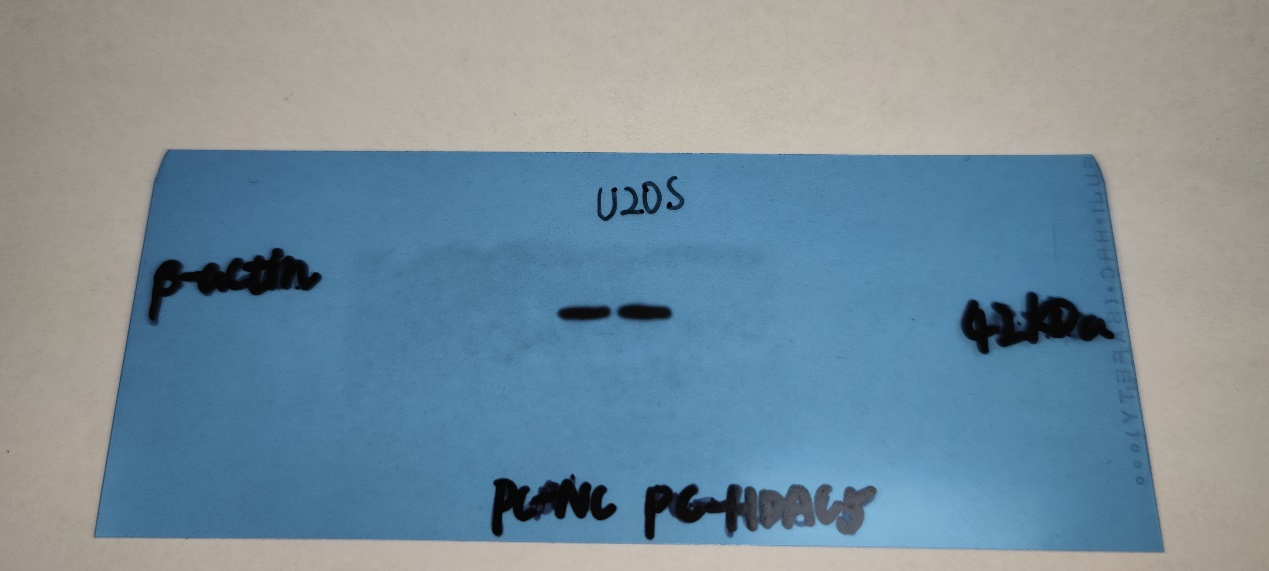


**Figure 5A**

**H3ac**


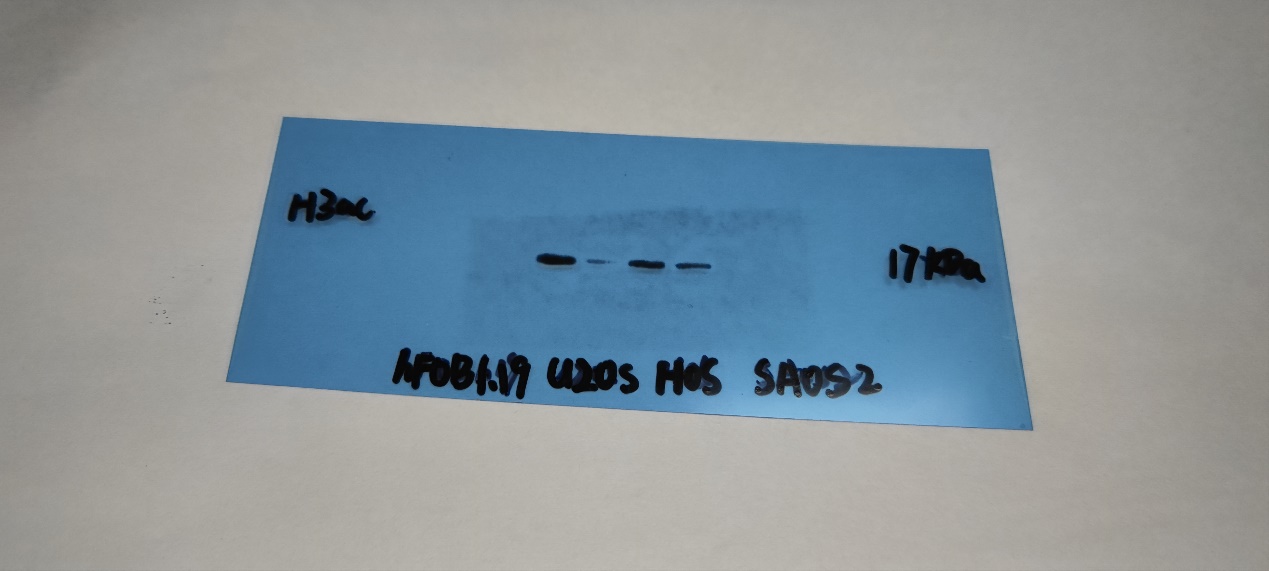


**Figure 5A**

**H4ac**


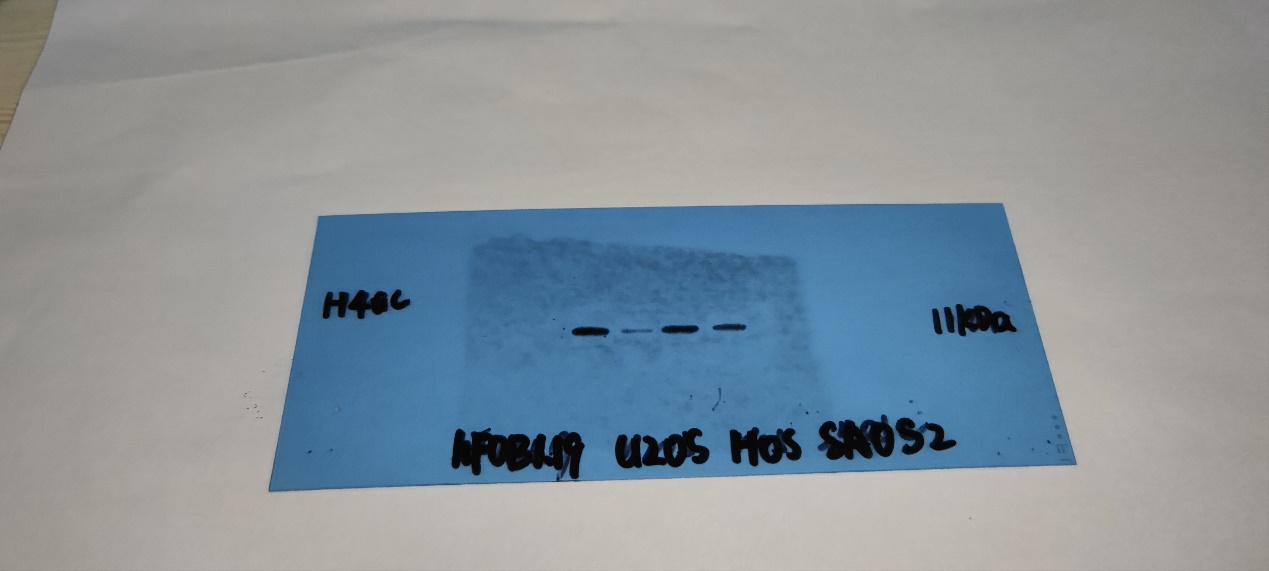


**Figure 5A**

**β-actin**


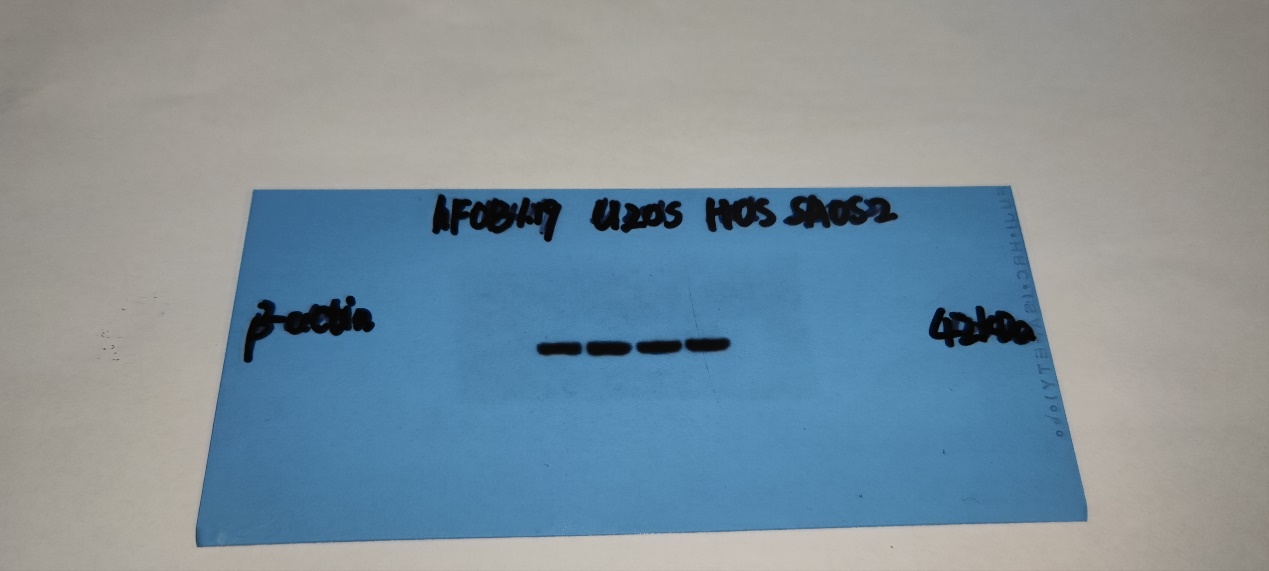

Supplement: Supplementary file 5 — Western blots [file 41420_2022_926_MOESM5_ESM.docx]
